# Aberrant Gene Expression and Sexually Incompatible Genomic Imprinting in Oocytes Derived from XY Mouse Embryonic Stem Cells *In Vitro*

**DOI:** 10.1371/journal.pone.0058555

**Published:** 2013-03-05

**Authors:** Mai Nitta, Masanori Imamura, Yu Inoue, Yasuo Kunitomo, Zachary Yu-Ching Lin, Takuya Ogawa, Keiichiro Yogo, Norihiro Ishida-Kitagawa, Noritaka Fukunaga, Hideyuki Okano, Eimei Sato, Tatsuo Takeya, Jun Miyoshi

**Affiliations:** 1 Graduate School of Biological Sciences, Nara Institute of Science and Technology, Nara, Japan; 2 Department of Molecular Biology, Osaka Medical Center for Cancer and Cardiovascular Diseases, Osaka, Japan; 3 Department of Physiology, Keio University, Tokyo, Japan; 4 Laboratory of Animal Reproduction and Physiology, Shizuoka University, Shizuoka, Japan; 5 Laboratory of Animal Reproduction, Tohoku University, Miyagi, Japan; Baylor College of Medicine, United States of America

## Abstract

Mouse embryonic stem cells (ESCs) have the potential to differentiate into germ cells (GCs) *in vivo* and *in vitro*. Interestingly, XY ESCs can give rise to both male and female GCs in culture, irrespective of the genetic sex. Recent studies showed that ESC-derived primordial GCs contributed to functional gametogenesis *in vivo*; however, *in vitro* differentiation techniques have never succeeded in generating mature oocytes from ESCs due to cryptogenic growth arrest during the preantral follicle stages of development. To address this issue, a mouse ESC line, capable of producing follicle-like structures (FLSs) efficiently, was established to investigate their properties using conventional molecular biological methods. The results revealed that the ESC-derived FLSs were morphologically similar to ovarian primary-to-secondary follicles but never formed an antrum; instead, the FLSs eventually underwent abnormal development or cell death in culture, or formed teratomas when transplanted under the kidney capsule in mice. Gene expression analyses demonstrated that the FLSs lacked transcripts for genes essential to late folliculogenesis, including gonadotropin receptors and steroidogenic enzymes, whereas some other genes were overexpressed in FLSs compared to the adult ovary. The E-Cadherin protein, which is involved in cell-to-cell interactions, was also expressed ectopically. Remarkably, it was seen that oocyte-like cells in the FLSs exhibited androgenetic genomic imprinting, which is ordinarily indicative of male GCs. Although the FLSs did not express male GC marker genes, the DNA methyltransferase, *Dnmt3L*, was expressed at an abnormally high level. Furthermore, the expression of sex determination factors was ambiguous in FLSs as both male and female determinants were expressed weakly. These data suggest that the developmental dysfunction of the ESC-derived FLSs may be attributable to aberrant gene expression and genomic imprinting, possibly associated with uncertain sex determination in culture.

## Introduction

Oocyte development in mammals is controlled spatiotemporally in concert with folliculogenesis. In the female genital ridge, post-migratory primordial germ cells (PGCs) differentiate into primitive oocytes and form nascent follicular structures called primordial follicles. Follicles are the functional units of oogenesis, and at the primordial stage, they consist of a single oocyte surrounded by a layer of squamous pre-granulosa cells [1]. The primordial follicles then become primary follicles as the surrounding somatic cells differentiate into cuboidal granulosa cells [2]. In these follicles, oocyte growth and granulosa cell proliferation are highly coordinated in order to form secondary follicles. Folliculogenesis then continues in particular follicles upon gonadotropin stimulation, which leads to the formation of an antral follicle consisting of a fluid-filled cavity surrounded by multiple layers of granulosa cells [1]. Finally, the follicles begin to mature into Graafian follicles ready for ovulation and fertilization. Throughout this process, oocytes undergo various developmental programs including growth arrest and reinitiation, meiosis and epigenetic reprogramming [3], [4].

To understand the mechanisms underlying multistep folliculogenesis, efforts have been made to design simple *in vitro* experimental systems to allow extensive investigations using molecular and cellular biological approaches. Attempts at achieving *in vitro* folliculogenesis for the production of fertile and developmentally competent oocytes in culture are of long-standing [5]. Following the cultivation of neonatal ovarian tissues, live offspring were obtained from primordial follicles grown *in vitro* [6], [7]. Viable offspring have also been produced from isolated primary follicles [8] and from isolated secondary follicles by means of a three-dimensional culture system that mimicked the *in vivo* environment for oocyte growth and maturation [9]. Even fetal germ cells (GCs) have been persuaded to produce fertilizable mature oocytes through cultivation [10], although the full-term development of pups was not achieved. However, combining *in vitro* culture with nuclear exchange techniques [11] or *in vivo* transplantation [12] has succeeded in producing live offspring from fetal GCs.

Meanwhile, the focus has been on pluripotent stem cells as an alternative source for reconstructing GC development *in vitro* [13], [14]. With respect to male GCs, mouse embryonic stem cells (ESCs) can differentiate to produce sperm cells in culture or after transplantation in mice [15], [16]. Recently, it was also demonstrated that ESC-derived PGCs are capable of contributing to functional gametogenesis and normal offspring [17], [18]. By contrast, fertilizable and developmentally competent oocytes have not yet been obtained via the *in vitro* differentiation of pluripotent stem cells. In 2003, follicle-like structures (FLSs) containing oocyte-like cells were reported to originate from both XY and XX mouse ESCs *in vitro* [19]. These structures expressed some follicle marker genes and exhibited parthenogenesis-like development, but antral follicle formation was not reported. Subsequent studies have also demonstrated FLS differentiation from XY and XX mouse ESCs [20], [21], [22], [23], although the FLSs had the appearance of primary-to-secondary follicles and antrum formation was not observed. In other studies, oocyte-like cells were generated but FLSs were not formed [22], [24], [25], [26], [27], [28]. Thus, it appears that there are crucial obstacles to the development of folliculogenesis from pluripotent stem cells in culture, especially beyond the secondary follicle stage [14]. Unfortunately, the low efficiency of FLS differentiation has prevented detailed investigations into why ESC-derived FLSs are unable to complete folliculogenesis.

In this study, we established a mouse ESC line capable of the highly efficient production of FLSs, which could be gathered in sufficient numbers for conventional molecular biological experiments. Because most of the previous trials have investigated XY mouse ESCs for *in vitro* oocyte/FLS differentiation studies, we also utilized XY mouse ESCs as starting cell materials. Gene expression analyses were performed and the genomic imprinting status of the FLSs was examined. The results helped to identify the molecular aberrations that distinguished the ESC-derived FLSs from normal ovarian follicles. This study provides an important benchmark of pluripotent stem cell-derived FLSs for the faithful recapitulation of folliculogenesis in culture.

## Results

### FLS formation by *in vitro* differentiation of mouse ESCs

First, three different mouse ESC lines (RW-4, E14 and B6) were examined for their ability to differentiate into FLSs *in vitro*. All of them were commercially available and known to be germline-competent. Each ESC line was cultured under LIF/feeder cell-free conditions and the number of FLSs that emerged was counted. No FLSs were observed in the B6 ESC culture; however, the RW-4 and E14 ESCs produced a small number of FLSs (around zero to six FLSs per 60 mm dish). Since the efficiency of FLS formation was not sufficient for detailed investigations, we attempted to obtain subclones of RW-4 ESCs with a higher competence of FLS production. To this end, the Δ*PE-Oct4pro-neo-IRES-GFP* reporter plasmid was introduced into RW-4 ESCs, as described in a previous study [19]. Eight neomycin-resistant ESC clones were selected; however, GFP fluorescence was not observed.

**Figure 1:**
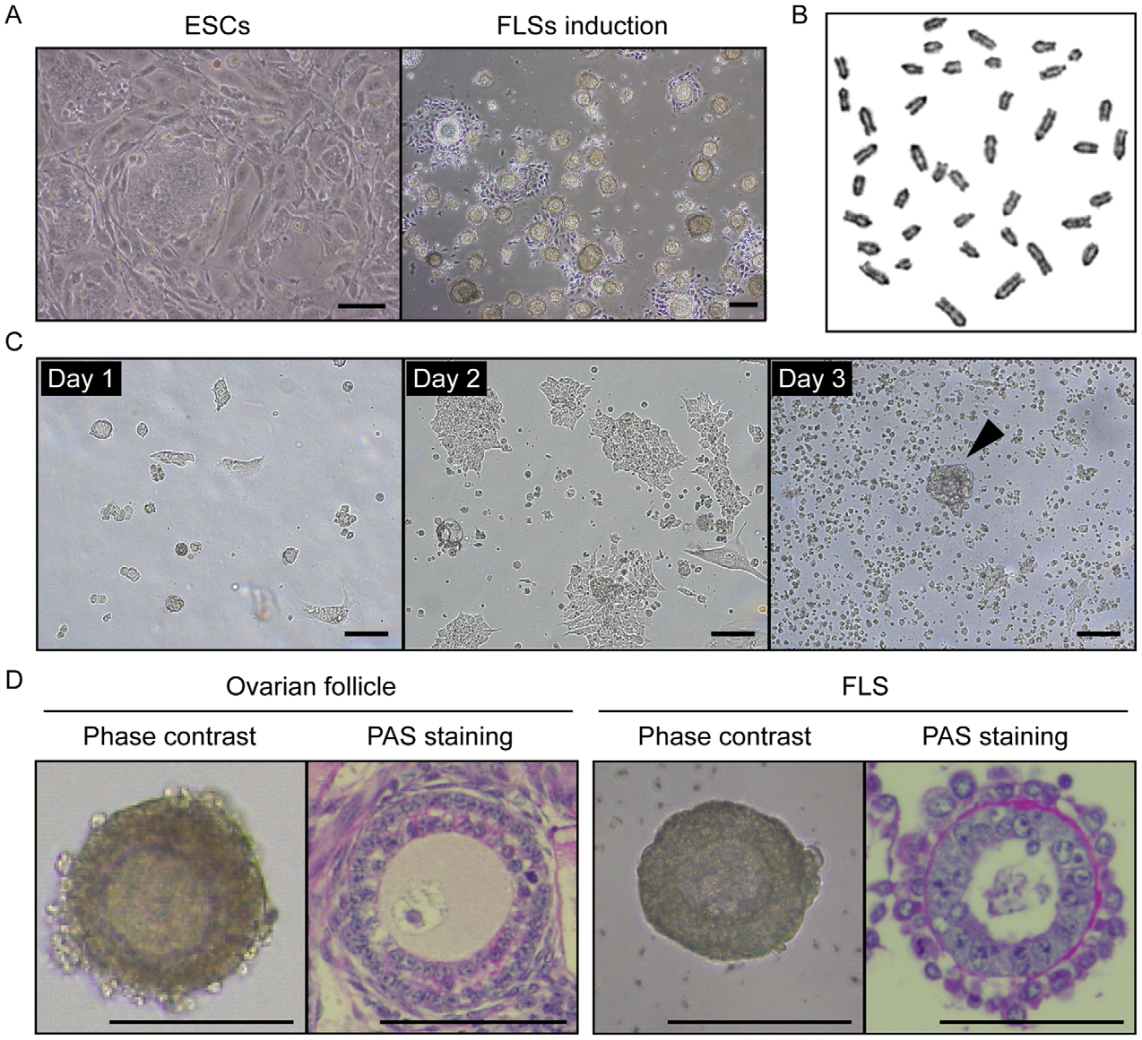
Morphology of mouse ovarian follicles and ESC-derived FLSs. (A) Morphology of undifferentiated clone-G ESCs on feeder cells (left side) and after FLS induction (right side). Scale bar, 100 μm. (B) G-banding chromosome analysis of clone-G ESCs demonstrating the normal 40, XY karyotype. (C) Morphology of differentiating clone-G ESCs during 3 days of FLS derivation culture. Arrowhead indicates an emerged FLS. Scale bar, 100 μm. (D) Mouse ovarian secondary follicle and clone-G ESC-derived FLS. Images of Periodic acid-Schiff (PAS) staining are shown on the right of each panel. Scale bar, 100 μm.

The subclones were able to form FLSs upon differentiation, similar to their parental ESCs. One ESC subclone (clone-G) generated nearly 100-fold more FLSs (246 to 262 FLSs per 60 mm dish) than the parental RW-4 ESCs or the other subclones (Fig. 1A) while retaining a normal karyotype (Fig. 1B). The floating FLSs mainly appeared after 3 days of differentiation culture (Fig. 1C). The FLSs were morphologically and structurally similar to ovarian secondary follicles, large oocyte-like cells in the center with surrounding layers of granulosa-like cells, basal lamina and theca-like cells in the outermost layer (Fig. 1D). Thus, clone-G ESCs were used in further experiments.

### BMP4 and BMP8b promote FLS formation from ESCs

**Figure 2:**
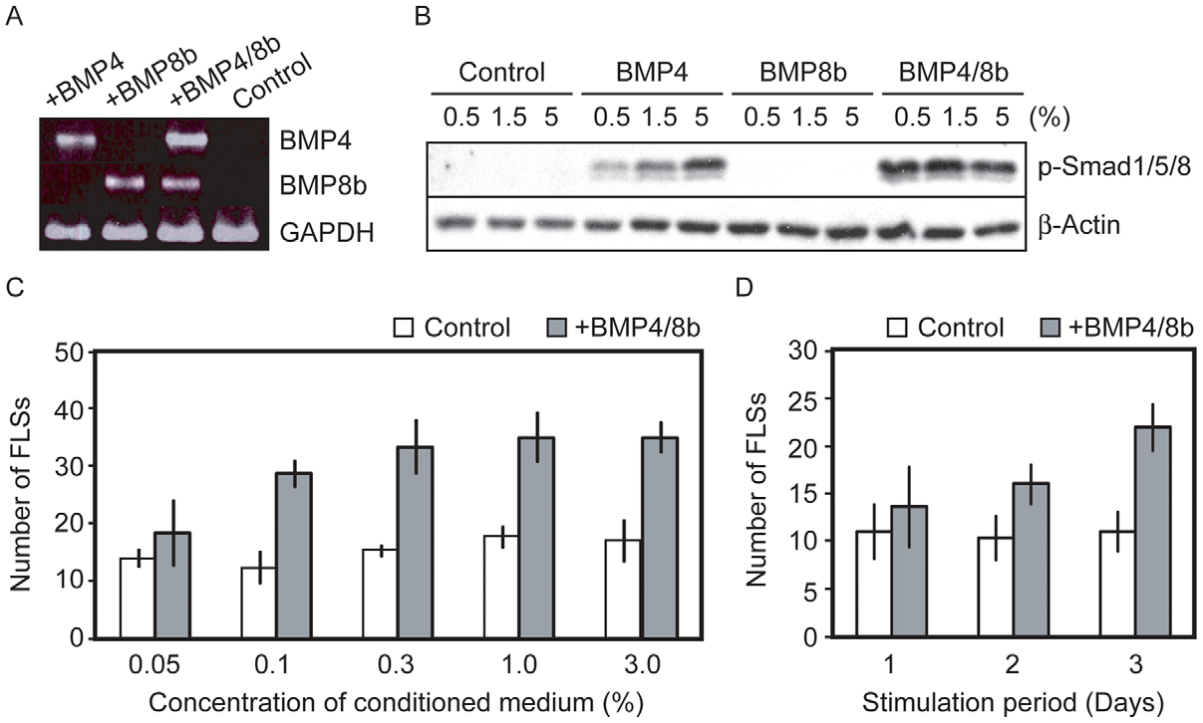
Facilitatory effect of BMP4/8b on FLS differentiation from ESCs. (A) Generation of BMP4/8b-expressing cell lines. NIH3T3 cells were modified by retroviral transduction of BMP4 and/or BMP8b ORFs, and RT-PCR was performed to confirm transgene expression. GAPDH was analyzed as an internal control. The supernatants were collected and utilized as conditioned media. (B) Western blot analysis of phosphorylated Smad1/5/8 proteins (p-Smad1/5/8). MC3T3-E1 cells were incubated with conditioned media containing BMP4 and/or BMP8b at final concentrations of 0.5%, 1.5% and 5%. β-Actin was analyzed as an internal control. (C) Concentration-dependent effects of BMP4/8b conditioned medium on FLS formation. Clone-G ESCs were cultivated under FLS differentiation conditions in the presence of different concentrations of conditioned medium (0.05% to 3.0%) for 3 days. The number of FLSs formed was counted on day 3. (D) Incubation duration-dependent effects of BMP4/8b conditioned medium on FLS formation. Clone-G ESCs were cultivated under FLS differentiation conditions with different time periods of stimulation by the conditioned medium. The final concentration of the conditioned medium was 3%. The number of FLSs formed was counted on day 3.

Previous studies reveal that bone morphogenetic protein 4 (BMP4) and BMP8b signaling pathways enhance PGC specification from ESCs [15], [29], [30]. In addition, BMP4 also promotes primordial-to-primary follicle development [31], [32]. Thus, we next investigated the effect of BMP4 and BMP8b on FLS formation from clone-G ESCs. BMP4 and BMP8b were expressed exogenously in NIH3T3 cells alone, or in combination, via retroviral transduction (Fig. 2A), and their supernatants were collected to be utilized as conditioned media. The phosphorylation of Smad proteins was most prominent when incubated with the conditioned medium prepared from BMP4/8b-double expressing NIH3T3 cells (Fig. 2B). In addition, the number of FLSs increased up to two to three times compared to the control culture. Interestingly, the enhanced FLSs formation was observed in a conditioned medium, dose-dependent manner (Fig. 2C). When the effective duration of BMP4/8b stimulation was assessed, the results demonstrated that longer treatment led to higher FLS formation (Fig. 2D). Taken together, the data confirmed that our FLS formation system reflected the correct BMP responsivity expected of GC development.

### ESC-derived FLSs do not contribute to normal folliculogenesis

**Figure 3:**
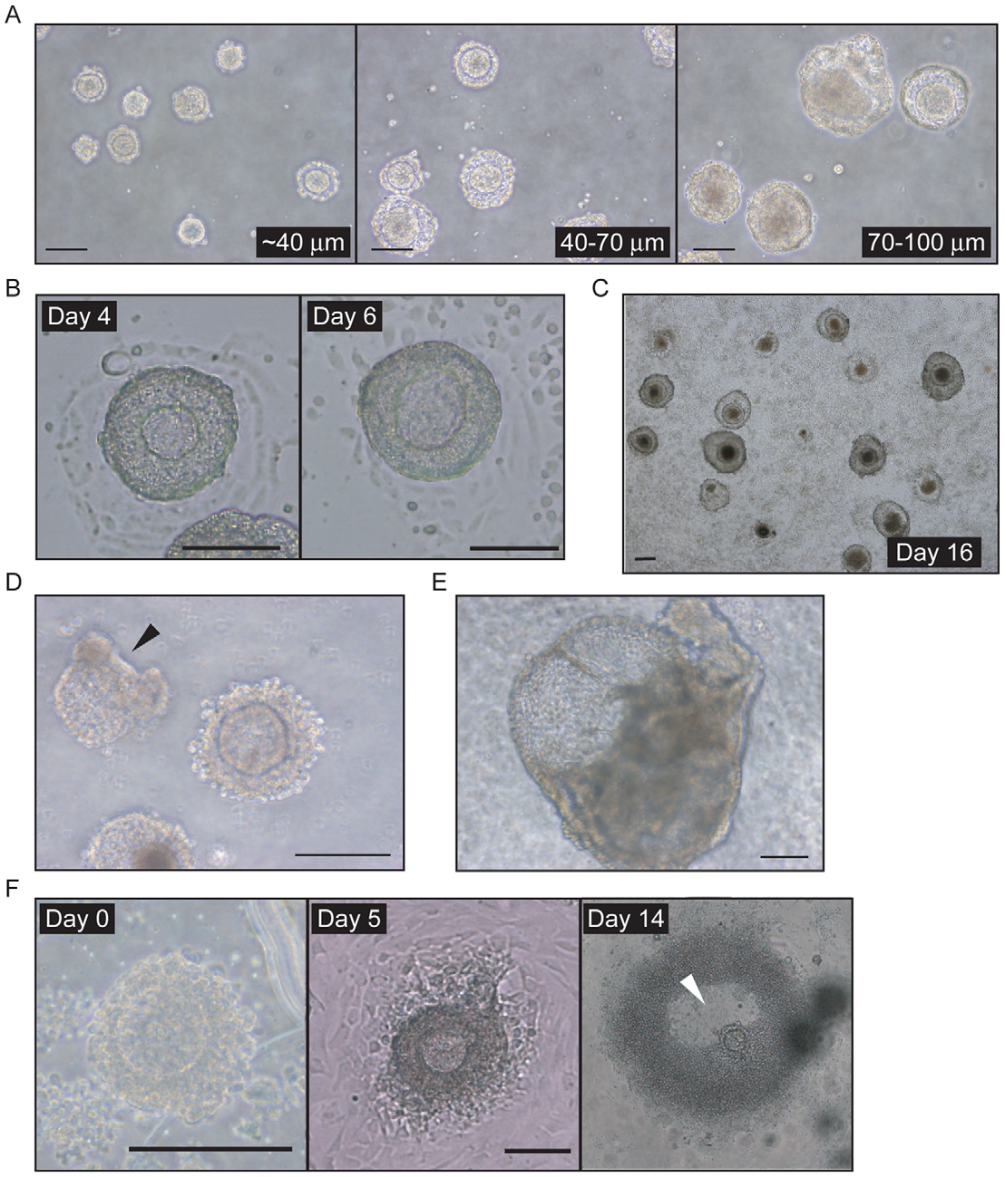
*In vitro* maturation culture of ESC-derived FLSs. (A) Size variation of FLSs emerged in 3-day differentiation cultures of clone-G ESCs. The FLSs were separated through membrane filters with three different pore sizes (40, 70, 100 μm). Scale bar, 100 μm. (B) Prolonged culture of FLSs. Following the 3-day differentiation culture, floating FLSs were collected and transferred to another dish for further cultivation. Scale bar, 100 μm. (C) Wide-field view of prolonged FLS culture on day 16. Scale bar, 100 μm. (D) FLSs in prolonged culture on day 6. Arrowhead indicates a collapsed FLS, which underwent abnormal development. Scale bar, 100 μm. (E) FLSs developing abnormally in collagen gels emerged after 10 days of culture. Scale bar, 100 μm. (F) Maturation culture of ovarian follicles using the ‘one-drop, one-follicle’ method. Secondary follicles isolated from mouse ovaries were cultivated for 14 days. Arrowhead indicates an antrum. Scale bar, 100 μm.

Although ESC-derived FLSs exhibited size variation in 3-day differentiation culture, FLSs with an antrum were not observed (Fig. 3A). Floating FLSs were collected from a 3-day differentiation culture and transferred into another dish for long-term culture. The FLSs attached to the new culture dishes and the granulosa-like cells started to expand (Fig. 3B, C). However, no FLSs were observed with an antrum; instead, the structures underwent abnormal development or cell death (Fig. 3D). This was also the case when the FLSs were cultured in another type of media such as αMEM or Waymouth's medium (data not shown). FLS cultures were tested further in collagen gels, which resulted in the degradation of the FLSs and abnormal development, which sometimes included beating cells (Fig. 3E).

**Figure 4:**
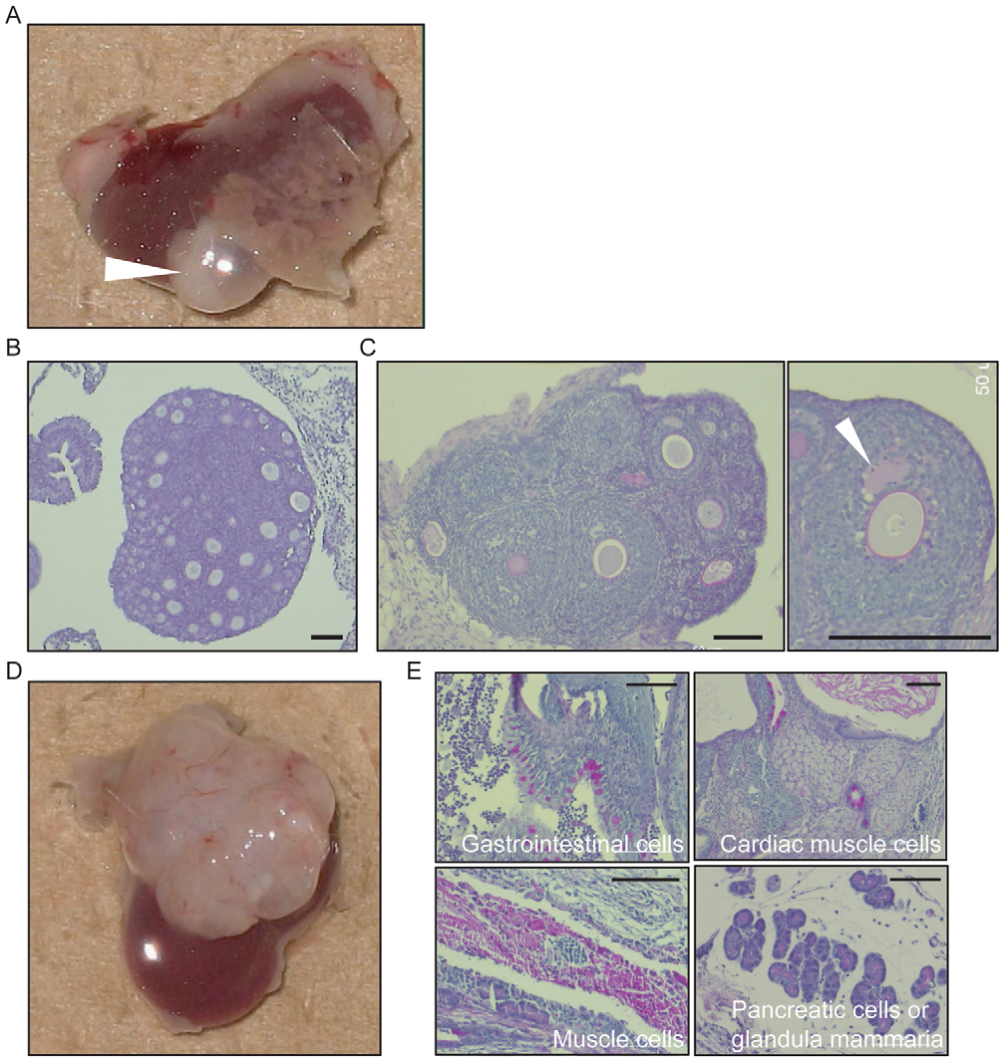
Transplantation of ESC-derived FLSs under the kidney capsule of SCID mice. (A) Transplantation of newborn mouse ovary under the kidney capsule of a SCID mouse. Arrowhead indicates the grafted ovary 2 weeks after transplantation. (B) PAS staining of neonatal ovary sections before transplantation. No antral follicles were observed. Scale bar, 100 μm. (C) PAS staining of the grafted ovary sections. The progression of folliculogenesis was observed in the graft as shown by antral cavity formation (arrowhead). Scale bar, 100 μm. (D) Teratoma formation following transplantation of clone-G ESC-derived FLSs under the kidney capsule of SCID mouse. (E) Sections of the teratoma derived from transplanted FLSs. The representative images show sections with gastrointestinal cells, cardiac muscle cells, muscle cells and cells that resembled pancreatic cells or glandula mammaria. Scale bar, 100 μm.

Mouse ovarian primary follicles can undergo folliculogenesis to antral follicles *in vitro* in the ‘one-drop, one-follicle system’, and these *in vitro*-matured follicles are capable of being fertilized (Fukunaga and Sato, unpublished data). Thus, the ‘one-drop’ method was used to determine whether ESC-derived FLSs were able to undergo an appropriate maturation process. After 14 days in culture, ovarian follicles underwent folliculogenesis and antrum formation (Fig. 3F). However, the clone-G ESC-derived FLSs did not form an antrum and were eventually degraded as before (data not shown). To assess the folliculogenesis potential of FLSs further, clone-G ESC-derived FLSs were transplanted under the kidney capsule of SCID mice. When the neonatal ovary was transplanted, folliculogenesis was attained as shown by the formation of antral follicles (Fig. 4A–C). However, the FLSs did not undergo normal folliculogenesis, but instead formed teratomas composed of various tissues (Fig. 4D, E).

### ESC-derived FLSs lack gonadotropin receptors and steroidogenic enzymes

**Figure 5:**
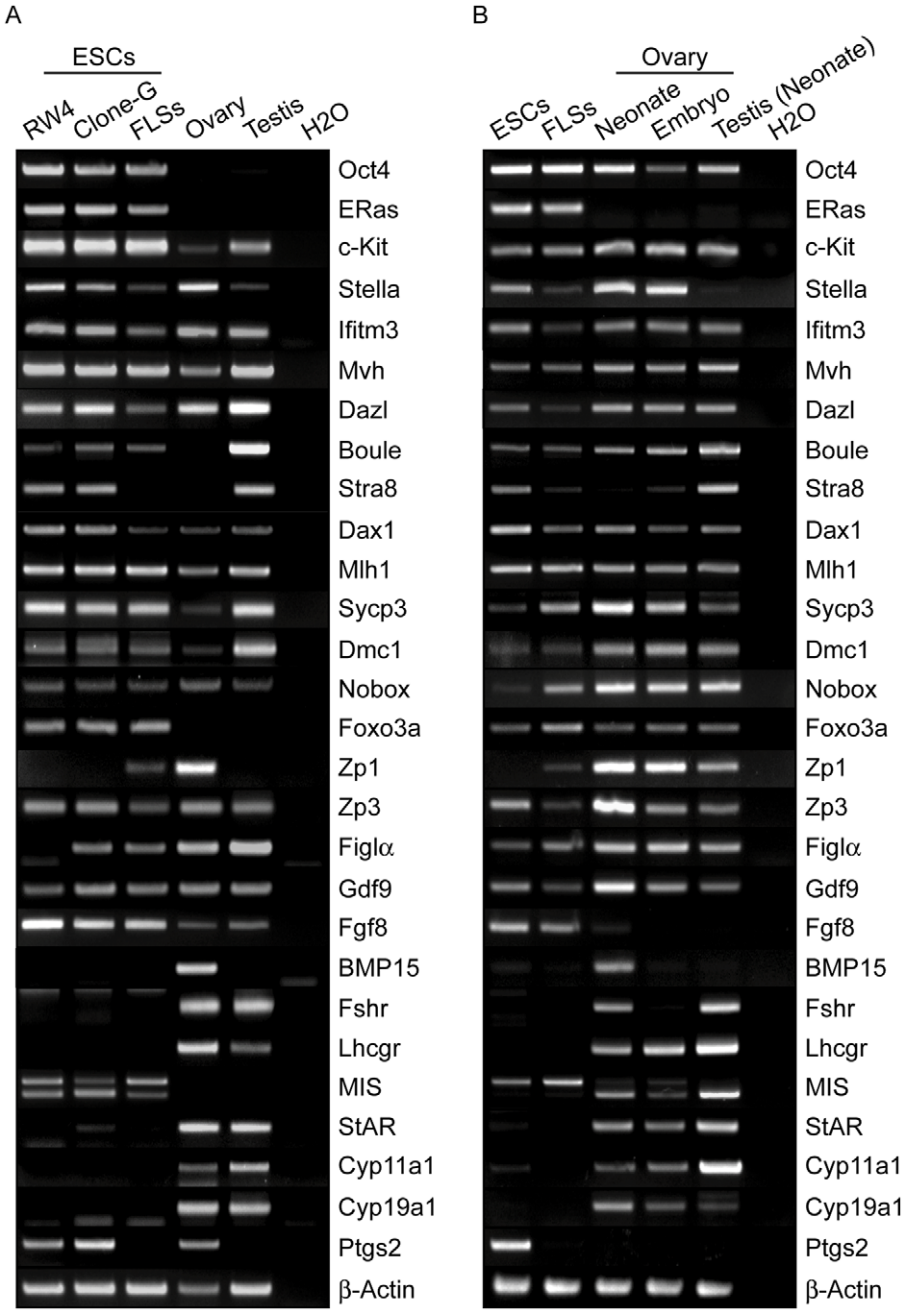
Gene expression profiles of ESC-derived FLSs. (A) The expression of GCs and folliculogenesis marker genes was examined by semi-quantitative RT-PCR analysis. Only representative images from three escalated cycles are presented. Total RNA was prepared from RW-4 ESCs, clone-G ESCs, clone-G ESC-derived FLSs (day 3), adult mouse ovary and testis. *β-Actin* was analyzed as an internal control and water was used as a negative control. (B) Gene expression in clone-G ESC-derived FLSs (day 3) was compared to those in embryonic (E19.0) and neonatal (P5) ovaries and in neonatal testis by semi-quantitative RT-PCR analysis as shown in (A).

Next, we examined the gene expression profiles of GC and follicle markers in the ESC-derived FLSs. Total RNA was prepared from the FLSs and subjected to RT-PCR analysis. In general, most of the genes examined were expressed in the FLSs, although the expression profiles differed from the adult ovary (Fig. 5A). The expression of some genes (*Stella*, *Ifitm3*, *Dazl*, *Nobox*, *Zp1*, *Zp3* and *Figlα*) seemed lower in the FLSs than in the ovary. On the other hand, the FLSs showed higher expression of *Oct4*, *ERas*, *c-Kit*, *Boule*, *Sycp3*, *Dmc1* and *Foxo3a* compared to adult ovaries. Remarkably, we found that FLSs were depleted of transcripts essential for late folliculogenesis. For example, among oocyte-secreted growth factors, *Gdf9* and *Fgf8* transcripts were abundant in FLSs, but *BMP15* was undetectable. Furthermore, FLSs lacked the gonadotropin receptors (*Fshr*, *Lhcgr*) that mediate follicle stimulating hormone (FSH) and luteinizing hormone (LH) signals to instruct antrum formation [33]. By contrast, the FLSs expressed abnormally high levels of *MIS*, which attenuates the FSH-dependent growth of preantral follicles [32]. In addition, the expression of genes associated with steroidogenesis (*StAR*, *Cyp11a1*, *Cyp19a1*, *Ptgs2*) was not observed in the FLSs.

We further compared the gene expression in FLSs with those in embryonic (E19.0) and neonatal (P5) mouse ovaries, and noticed the lack of gonadotropin receptors and steroidogenesis-associated genes in FLSs again (Fig. 5B). Additionally, abnormally high expression of *ERas* and *Fgf8* was also observed in FLSs. On the other hand, unlike adult ovary, the expression of *Oct4*, *Boule*, and *Foxo3a* was detected in both embryonic and neonatal ovaries in a similar fashion to FLSs. When compared with neonatal ovary, the gene expression profile in FLSs seemed to be closer to that in embryonic ovary as represented by *Stra8* and *Zp3* expression, suggesting that oocyte-like cells in FLSs might stay in an immature stage of oogenesis.

### Ectopic overexpression of E-Cadherin in ESC-derived FLSs

**Figure 6:**
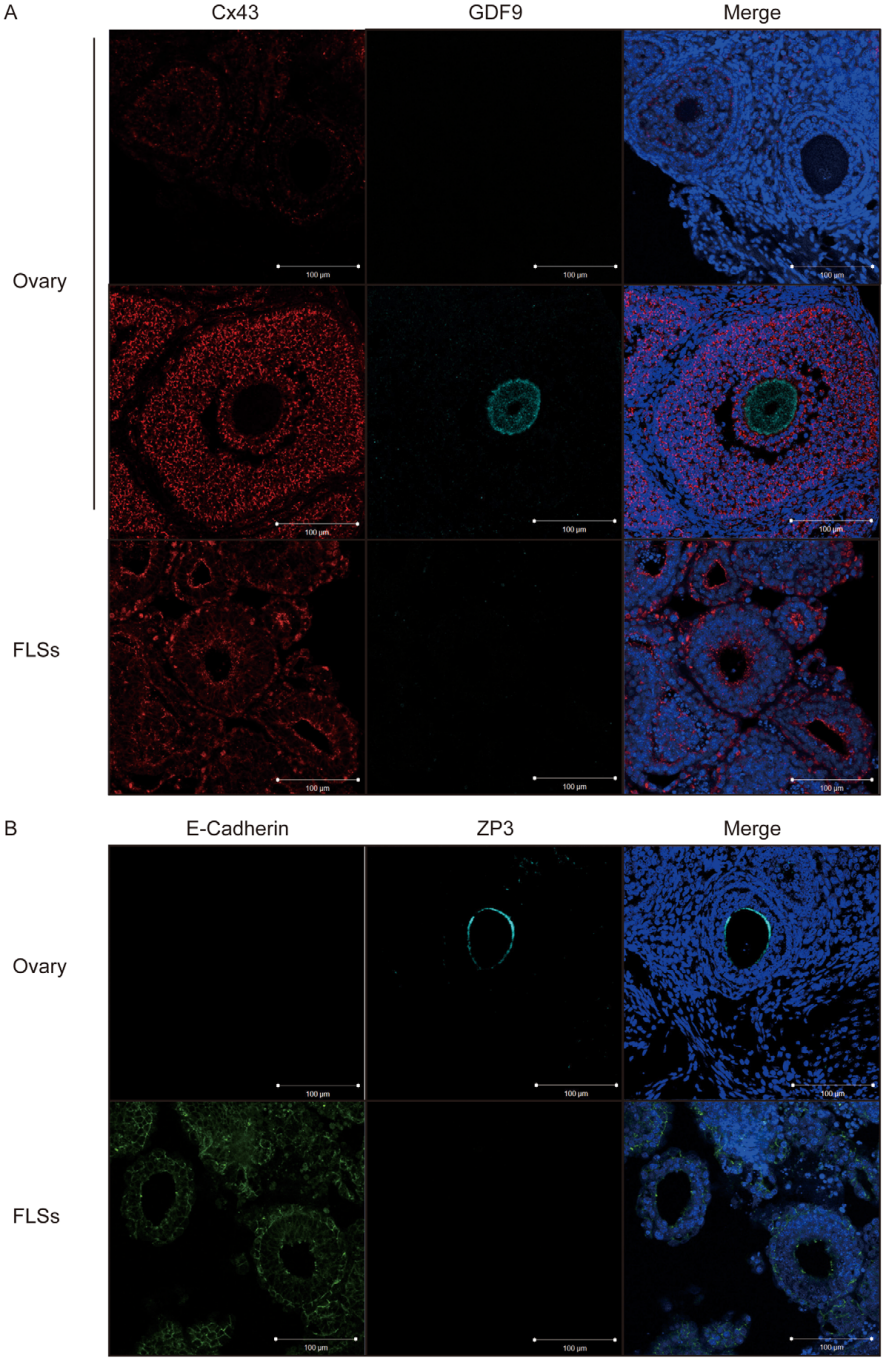
Immunohistochemistry of ESC-derived FLSs. (A) Immunostaining of Cx43 and GDF9 in neonatal ovary and clone-G ESC-derived FLSs. Nuclei were stained with Hoechst 33342. Scale bar, 100 μm. (B) Immunostaining of E-Cadherin and ZP3 proteins in neonatal ovary and FLSs. Nuclei were stained with Hoechst 33342. Scale bar, 100 μm.

To ensure the process of folliculogenesis, the appropriate cell-cell interactions are essential. Cell adhesion proteins were examined in FLSs using immunofluorescence microscopy. In the adult ovary, the gap junction protein, Cx43, was expressed weakly in the granulosa cells of secondary follicles; however, the expression intensified in early antral follicles (Fig. 6A). GDF9 protein was not detected in secondary follicles, whereas it was observed in early antral follicles. The expression of Cx43 seemed higher in the granulosa-like cells of the FLSs than in the granulosa cells of ovarian secondary follicles. On the other hand, no FLSs expressed GDF9 protein. Another cell adhesion molecule, E-Cadherin, was also examined. Although ovarian follicles did not express the protein at any stage, abnormal E-Cadherin expression was observed in FLSs, especially in the cells located in the outer layers of the structures (Fig. 6B). In addition, it turned out that ZP3 protein was detected in oocytes of ovarian secondary follicles but not in the oocyte-like cells of the FLSs, unlike the transcript, which was expressed in the FLSs, albeit at lower levels than in the adult ovary (Fig. 5).

### Androgenetic genomic imprinting in oocyte-like cells of ESC-derived FLSs

**Figure 7:**
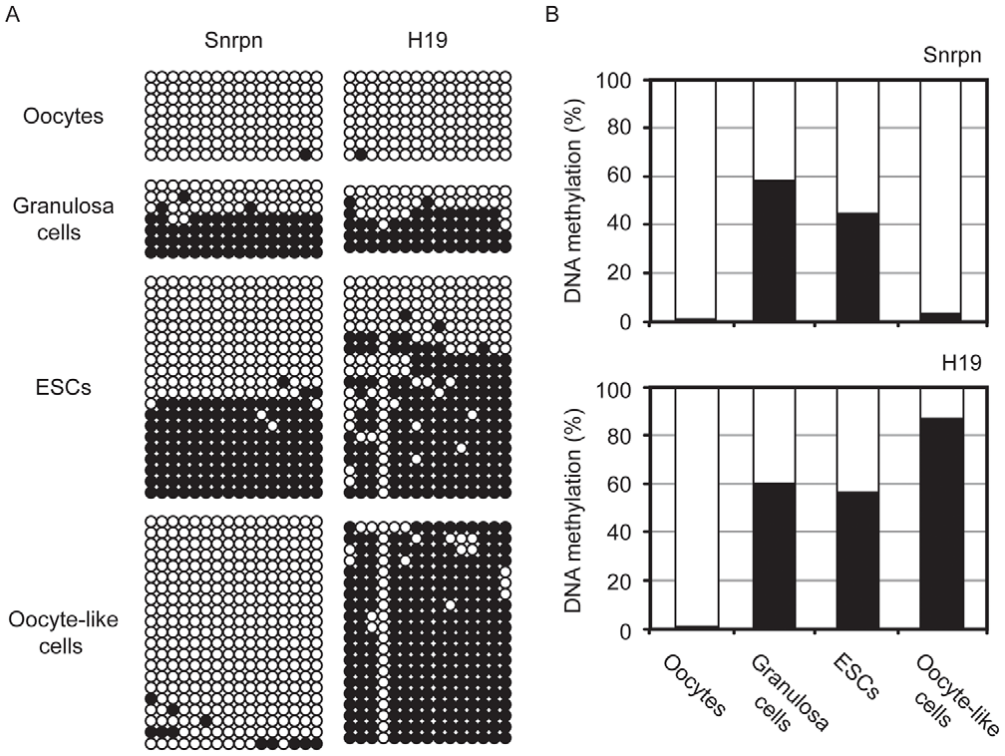
DNA methylation status of imprinted genes *Snrpn* and *H19.* (A) Bisulfite genomic sequencing was performed to examine the genomic imprinting status of DMRs in the maternal imprinted gene, *Snrpn*, and the paternal imprinted gene, *H19*. Open and closed circles represent unmethylated and methylated CpG dinucleotides respectively. Oocytes and granulosa cells were separated from 5-day-old ovarian secondary follicles. Similarly, oocyte-like cells were isolated from clone-G ESC-derived FLSs. Undifferentiated clone-G ESCs were also analyzed as the control. (B) Quantification of DNA methylation levels. The extent of CpG methylation was divided by the total number of CpG sites, based on the result shown in (A).

To ascertain whether female GC-specific epigenetic programs were imposed correctly on oocyte-like cells of the FLSs, bisulfite genomic sequencing of maternal (*Snrpn*) and paternal (*H19*) imprinted loci was performed. During mouse oogenesis, the differentially methylated region (DMR) of *Snrpn* remains unmethylated in neonatal oocytes and becomes gradually methylated from postnatal day 10 [34]. By contrast, the *H19* DMR remains in an unmethylated state throughout oogenesis. As a control, ovarian secondary follicles were collected from mice up to 5-days-old, and were separated into oocytes and granulosa cells. A total of 20 cells of each type were used for analysis. As expected, both *Snrpn* and *H19* remained unmethylated in oocytes while granulosa cells displayed the parental methylation pattern (Fig. 7A, B). Similar to granulosa cells, the undifferentiated clone-G ESCs retained parental genomic imprinting. However, oocyte-like cells isolated from ESC-derived FLSs exhibited demethylation of *Snrpn* concomitant with increased methylation of *H19*, indicative of a male GC-specific androgenetic imprinting pattern.

**Figure 8:**
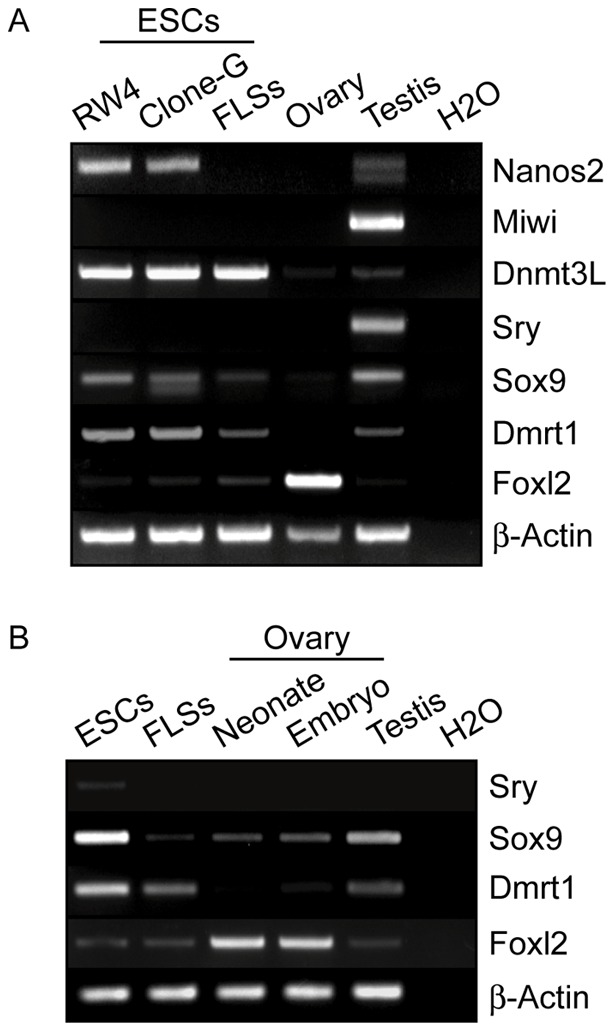
Expression of genes associated with sex determination in ESC-derived FLSs. (A) Semi-quantitative RT-PCR analysis was performed to examine the expression of male GC marker genes (*Nanos2*, *Miwi*, *Dnmt3L*) and sex determination factors of male (*Sry*, *Sox9*, *Dmrt1*) and female (*Foxl2*) gonads. Only representative images from three escalated cycles are presented. Total RNA was prepared from RW-4 ESCs, clone-G ESCs, clone-G ESCs-derived FLSs (day 3), adult mouse ovary and testis. *β-Actin* was analyzed as an internal control and water was used as a negative control. (B) Gene expression in clone-G ESC-derived FLSs (day 3) was compared to those in embryonic (E19.0) and neonatal (P5) ovaries and in neonatal testis by semi-quantitative RT-PCR analysis as shown in (A).

Considering the genomic imprinting status in FLS oocyte-like cells, the expression of genes involved in sex determination was investigated. Despite the androgenetic genomic imprinting, the male GC marker genes, *Nanos2* and *Miwi*, were not expressed in FLSs (Fig. 8); by contrast, FLSs highly expressed *Dnmt3L*, which is normally expressed preferentially in male GCs and is responsible for establishing paternal methylation imprints [35]. A previous study showed that the ectopic expression of *Sry*, the most potent male determinant, produced a partial androgenetic imprinting pattern in XX GCs [36]; however, no *Sry* expression was observed in FLSs. On the other hand, we found that other male determination factors, *Sox9* and *Dmrt1*, were expressed ectopically in FLSs although the expression was weak. Intriguingly, FLSs also expressed the female gonad determination factor, *Foxl2*, albeit at much lower levels than in the ovary. Together, these results suggested that sex determination would be perturbed in ESC-derived FLSs.

## Discussion

**Figure 9:**
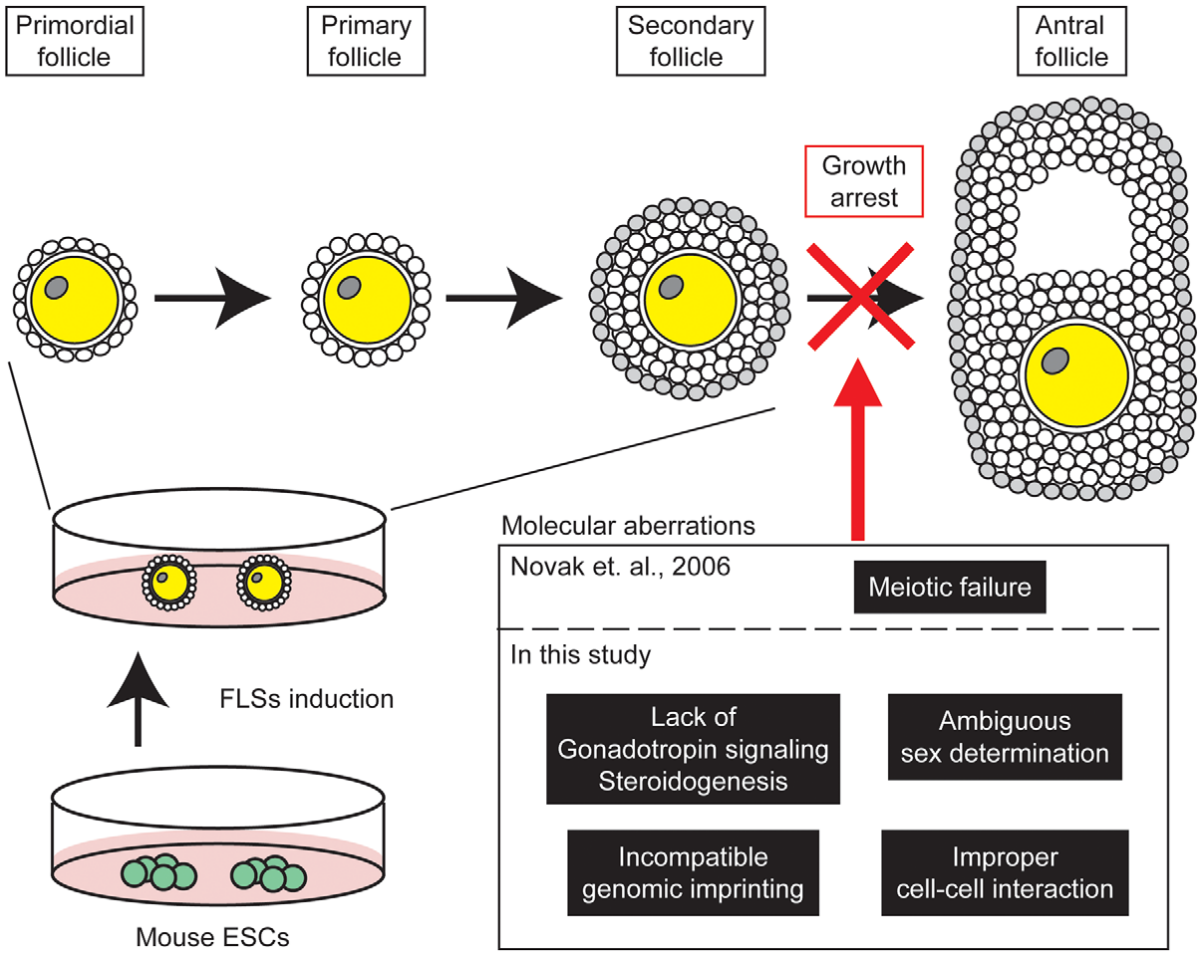
Model summarizing the molecular abnormalities in ESC-derived FLSs *in vitro*. Mouse ESCs can mimic folliculogenesis under *in vitro* differentiation culture conditions, but the progression is aborted before antrum formation. Previously, Novak *et al.* reported that ESC-derived FLSs do not form meiosis-specific chromosomal organization. In this study, we identified other novel molecular aberrations originating in FLSs, including a lack of gonadotropins and estrogen signaling, sexually incompatible genomic imprinting, ambiguous sex determination and abnormal cell-cell interactions, and our model suggests that a combination of these aberrations hinders the progression of normal folliculogenesis in ESC differentiation culture.

Despite increasing reports of oocyte-like cell differentiation from pluripotent stem cells *in vitro* [19], [20], [21], [22], [23], [24], [25], [26], [27], [28], [37], little is known about the underlying mechanisms prohibiting their normal development, owing to the low efficiency and reproducibility of differentiation. To date, only a few studies have focused on the abnormalities commonly observed in ESC-derived oocytes, demonstrating the absence of meiotic chromosomal organization and its associated proteins in mouse ESC-derived FLSs [20], [28], [38]. However, it is not enough simply to interpret folliculogenesis arrest; so to perform further detailed investigations, we established clone-G ESCs which were capable of forming FLSs at high efficiency. As shown in the previous studies using other XY and XX ESC lines, the clone-G ESCs-derived FLSs did not grow beyond the secondary follicle stage (Fig. 3). However, it is worth noting that the ESCs required only 3 days for FLS derivation in the culture system described in the present study, which is, to our knowledge, the shortest period known to induce oocyte-like cells or FLSs. Using this ESC clone, novel aberrations in gene expression and genomic imprinting were identified in the derivative FLSs (Fig. 9). Thus, the clone-G ESCs facilitated the diagnostic analyses of FLS properties using conventional molecular biological approaches.

Remarkably, the gene expression profiles revealed that FLSs would be unresponsive to gonadotropin signals as they lacked gonadotropin receptors for FSH and LH (Fig. 5). In normal ovarian follicles, the FSH receptor is expressed in granulosa cells while the LH receptor is expressed in theca cells [33]. Folliculogenesis proceeds independently of FSH and LH signaling until the secondary follicle stage, after which time it becomes dependent on FSH/LH signaling [5], [39], [40]. In the absence of FSH and LH signaling, follicular development stalls prior to the antral and preovulatory stages, respectively.

In addition to the lack of gonadotropin receptors, the expression of steroidogenesis-associated genes was also depleted in FLSs, suggesting that FLSs may exhibit defective estrogen production. Similar to the role played by FSH, autocrine estrogen is essential for folliculogenesis beyond the antral follicle stage [41]. This lack of gonadotropin receptors and steroidogenic enzymes provides an obvious explanation for the arrested folliculogenesis in FLSs, which never form an antrum. Furthermore, normal cell-cell communication is vital for folliculogenesis as oocyte development is concurrent with, and interdependent on, that of the surrounding granulosa and theca cells [42]. Since E-Cadherin, which is not normally expressed in ovarian follicles, was expressed ectopically in the granulosa-like cells of FLSs (Fig. 6), atypical cell-cell interactions might be a further problematic factor in ESC-derived folliculogenesis.

On the other hand, a previous study showed that ESC-derived oocytes did not develop past the primary follicle stage even when co-aggregated with newborn ovarian cells and transplanted under the kidney capsule [28], suggesting that oocyte-like cell-autonomous defects also block further folliculogenesis. With respect to oocyte-like cells of the FLSs in the current study, an unexpected genomic imprinting error was identified in which these cells acquired an androgenetic imprinting pattern instead of a female GC pattern (Fig. 7), despite the fact that there was no detectable *Sry* expression in FLSs (Fig. 8). XY or ectopic XX oocytes acquire female-type genomic imprinting during oogenesis *in vivo*. For example, in sex-reversed mice with an *Sry* deletion, XY ‘female’ GCs exhibited *H19* hypomethylation as well as the correct maternal imprint of *Peg3* [36]. Even ‘testicular eggs’ in XX-XY chimeric mice underwent maternal imprinting, except for *Snrpn* which remained hypomethylated [43].

By contrast, the situation seems different in culture. Male PGC-derived embryonic germ cells (EGCs) displayed higher DNA methylation of the *Igf2*-*H19* locus than did female EGCs [44]. Importantly, this occurred independently of the *Sry* gene or the cellular phenotypic sex, since XX ‘male’ EGCs carrying an *Sry* transgene exhibited *H19* demethylation, while XY ‘female’ EGCs exhibited a higher level of DNA methylation [45]. Thus, in the absence of appropriate environmental signals, the sex chromosome constitution might act definitively to establish genomic imprinting in oocyte-like cells in culture. Besides this, it is possible that the culture conditions could bring about anomalous genomic imprinting, as reported previously for the demethylation of maternally imprinted genes (*Igf2r* and *Peg1*) and the increased methylation of *H19* in cultivated oocytes [46], [47], [48].

Additionally, it is likely that the process of sex determination was perturbed in FLSs (Fig. 8). GC sex determination is implemented according to the sex of the surrounding gonadal environment [49]; for example, XY PGCs can differentiate into oocytes when placed in a fetal ovarian milieu whereas XX PGCs develop into spermatogonia in fetal testes [50], [51]. Gonadal sex is determined by certain pivotal transcription factors; *Sry*, *Sox9*, *Drmt1* in males [52] and *Foxl2* in females [53], and thus, their aberrant expression in FLSs might be associated with the androgenetic imprinting of oocyte-like cells. Therefore, could sexual incompatibility be a prevailing risk factor for folliculogenesis arrest? Male-to-female sex-reversed mice revealed not only that XY ovaries contained no preovulatory follicles, but also that the oocytes adhered poorly to cumulus cells [54]. Moreover, an impaired pairing of the sex chromosomes interrupts meiosis in XY oocytes [55], which are then incapable of supporting preimplantation embryonic development [56]. Indeed, the expression pattern of oocyte marker genes in clone-G ESCs-derived FLSs seemed similar to that in embryonic ovary rather than neonatal or adult ovaries, suggesting defects in oogenesis process in the oocyte-like cells (Fig. 5). Thus, although XY ESCs have been utilized to induce oocyte-like cells or FLSs in most studies, XX ESCs might be a better starting point. However, as with XY ESCs, using XX ESCs to produce oocyte-like cells or FLSs was unsuccessful in previous studies [19], [23], [25], [28]. In the current study, three XX ESC lines (BDft-1, BD129fc-1, 129fc-2) was also used to test FLS formation, although few FLSs were formed and the molecular characterization was impossible (data not shown). This might be due to genetic and epigenetic instability accompanying XX mouse ESC culture [57].

Following folliculogenesis arrest, the FLSs underwent abnormal development or cell death both *in vitro* and *in vivo* (Fig. 3, 4). It is unclear whether the abnormal development was due to parthenogenetic activation or dedifferentiation to the pluripotent state. Nevertheless, given the aberrant genomic imprinting in the oocyte-like cells (Fig. 7), the phenomenon could be attributed to cell autonomous defects in the oocyte-like cells themselves. A previous study demonstrated parthenogenesis-like development from FLSs in culture; however, they did not show antrum formation in FLSs [19], suggesting that the development might have arisen via irregular pathway(s) distinct from the normal folliculogenesis program. In the course of folliculogenesis, the vast majority of primordial follicles are abolished through atresia and only a small fraction of oocytes survive to initiate meiosis. This selection insures oocyte quality, which leads to the successful survival of the early embryo, the establishment and maintenance of the pregnancy and correct fetal development [33]. We speculate that current *in vitro* differentiation culture systems cannot recapitulate this selection process; therefore, ESC-derived FLSs easily deviate from *bona fide* folliculogenesis to abnormal development. To address this phenomenon, the molecular basis of the oocyte selection system should be of primary importance in future studies.

In conclusion, we believe that the current study provides helpful insights for future research. In general, clone-G ESCs exhibited gene expression profiles comparable to the parental RW-4 ESCs, although the transcription factor, *Figlα*, was expressed in clone-G ESCs but not in RW-4 ESCs (Fig. 5). FIGLα is essential for the initiation of folliculogenesis [58], therefore this could account, at least partly, for the efficient FLS formation from clone-G ESCs. The comparison of global gene expression between both the ESC lines investigated in this study should help to identify other candidate genes that commit pluripotent cells to folliculogenesis. In addition, we propose that FLSs and oocyte-like cells derived from pluripotent stem cells should be authenticated based on the expression of gonadotropin receptors, steroidogenesis enzymes, cell adhesion proteins and sex determination factors, as well as their genomic imprinting status (Fig. 9). Current methodology is not sufficient to induce complete *in vitro* folliculogenesis from pluripotent stem cells; however, future experiments designed to uncover the fundamental differences that exist between physiological and simulated folliculogenesis will help to unravel the processes controlling folliculogenesis step by step.

## Materials and Methods

### Ethics Statement

The experimental protocols involving mice were approved by the Animal Studies Committee at Nara Institute of Science and Technology, and performed according to the animal experimentation guidelines.

### ESC culture and FLS differentiation

The following ESC lines: RW-4 (from 129/SvJ male mice), E14TG2aIV (from 129/SvJ male mice: E14) and C57BL/6 (from C57BL/6 male mice: B6) were maintained in Dulbecco's modified Eagle's medium (DMEM) with high glucose (Nikken, Tokyo, Japan, CM4402) containing 20% fetal bovine serum (FBS; Invitrogen, Carlsbad, CA,10439-024), 0.1 mM non-essential amino acids (Invitrogen, 11140-050), 1X nucleotide solution, 2-mercaptoethanol (Wako, Osaka, Japan, 137-06862), and 1000 U/ml LIF (Millipore, Billerica, MA, ESG1107) on mitomycin C-treated STO cells at 37°C with 5% CO_2_. The 100X nucleotide solution was prepared by dissolving 80 mg of adenosine powder, 85 mg of guanosine powder, 73 mg of cytidine powder, 73 mg of uridine powder and 24 mg of thymidine powder in 100 ml sterile water. For differentiation into FLSs, ESCs were dissociated with 0.25% trypsin/0.02% EDTA (IBL, Gunma, Japan, 23315) and resuspended in ESC medium. The cells were incubated on 0.1% gelatin-coated dishes for 15 to 20 minutes to remove the STO feeder cells. Next, floating cells were collected and resuspended in FLS differentiation medium consisting of ESC medium with lower FBS (15%) and without LIF. The cells were plated at a density of 1×10^4^ cells/cm^2^ in uncoated tissue culture dishes (Iwaki, Tokyo, Japan, 3010–060) and were cultivated for 3 days without medium changes.

### Subcloning of RW-4 ESCs

A 4.9 kb region from the mouse *Oct4* promoter, of which the proximal enhancer (PE) region was removed using the Quick Change Site-Directed Mutagenesis Kit (Stratagene, CA, USA, 200518), was isolated. A *Neo* resistance gene from pMC1Neo3804 (Stratagene, 213201) and an IRES-hrGFP fragment from pIRES-hrGFP-1a (Stratagene, 240031) were introduced after the *Oct4* initiation codon in a pBLC cloning vector to create the plasmid, Δ*PE-Oct4pro-neo-IRES-GFP*. Then, this single-stranded plasmid was incorporated into RW-4 ESCs by electroporation. The ESCs were maintained in ESC medium for 2 days and then cultured in medium containing 0.3 mg/ml G418 for 7 days. A total of eight G418-resistant ESC colonies (clones A to H) were picked and expanded as subclones. Karyotyping of clone-G ESCs was entrusted to Nihon Gene Research Laboratories Inc. (Sendai, Japan).

### Morphological and histological analyses

For morphological observations, ovarian follicles were dissected mechanically from the ovaries of 9-day to 1-month-old C57BL/6 mice using 26-gauge needles. For Periodic acid-Schiff (PAS) staining, mouse ovaries or ESC-derived FLSs were fixed with Bouin's solution, embedded in paraffin, and sectioned at a thickness of 5 μm. After de-waxing, the sections were incubated with 0.5% periodic acid for 15 minutes. Following rinses with distilled water, the sections were incubated with Cold Schiff's Reagent (Wako, 039–14645) for 15 minutes and then with sulfurous acid solution, containing 0.54% sodium hydrogen sulfite and 0.045N HCl, three times for 3 minutes each. The sections were washed with running water for 10 minutes and finally were stained with hematoxylin for 1 minute.

### Preparation of BMP4/BMP8b conditioned medium

BMP4 and BMP8b open reading frames (ORFs) were cloned into pCX4puro and pCX4bsr retroviral vectors, respectively. NIH3T3 cells were infected with the viruses alone or in combination, and the cells were selected with 3 μg/ml puromycin and/or 10 μg/ml blasticidin for 2 weeks. To prepare conditioned medium, confluent infected NIH3T3 cells were cultivated in DMEM containing 10% FBS for 3 days, and the supernatants were collected as conditioned media. To assess the BMP4 and BMP8b bioactivities in the conditioned media, MC3T3-E1 cells were serum-starved for 3 hours and then incubated with each of the conditioned media for 30 minutes. Total proteins were extracted from the cells and used for Western blot analysis against phosphorylated Smad1/5/8 (1/1000, Millipore, AB3848).

### RT-PCR analysis

Total RNA was extracted using TRIzol reagent (Invitrogen, 15596–026), and cDNA was synthesized using SuperScript First-Strand Synthesis for RT-PCR (Invitrogen, 11904–018) according to the manufacturer's protocols. PCR amplifications were performed with Ex Taq Hot Start Version (TaKaRa, Shiga, Japan, RR006A). The primers used in this study are shown in Table S1. All experiments were performed semi-quantitatively at three escalation cycles, and only representative images are shown in the results.

### Immunofluorescence analysis

FLSs and adult mouse ovary were fixed with Bouin's solution, dehydrated and embedded in paraffin. Serial sections of 5-μm thickness were de-waxed with xylene, rehydrated in graded alcohol and washed with distilled water. For immunofluorescence analysis, the sections were subjected to antigen retrieval by autoclave at 105°C for 5 minutes in 1 M Target Retrieval Solution (Dako, Glostrop, Denmark, S2375), and blocked with 5% skim milk for 1 hour. Next, the sections were incubated with the following primary antibodies: rabbit anti-Cx43 polyclonal antibody (1/100, Sigma, MI, USA, C6219), goat anti-GDF9 polyclonal antibody (1/100, Santa Cruz, CA, USA, sc-12244), mouse anti-E-Cadherin monoclonal antibody (1/100, BD Biosciences, NJ, USA, 610182), goat anti-ZP3 polyclonal antibody (1/100, Santa Cruz, sc-23715) overnight at 4°C. The secondary antibodies were Alexa Fluor 488 goat anti-mouse IgG (1/250, Invitrogen, A-11029) and Alexa Fluor 555 goat anti-rabbit IgG (1/250, Invitrogen, A-21429). Nuclei were stained with 10 ng/ml Hoechst 33342.

### Follicle maturation culture

For ‘one-drop, one-follicle’ culture, 20 drops (20 μl) of αMEM (Invitrogen, 12561–056) supplemented with 5% FBS, 1X ITS (Invitrogen, 51300–044), and 200 mIU/μl FSH (Sigma, F4021) were placed in 60 mm dishes. The drops were covered with liquid paraffin (Nacalai Tesque, Kyoto, Japan, 26144–85) and incubated at 37°C for 4 hours. Then, ovarian follicles or FLSs were transferred into each drop one at a time. Half of the medium was exchanged every other day.

### Transplantation

Fifty FLSs derived from clone-G ESCs were embedded in 0.2% collagen gel (BD Biosciences, 354236) and transplanted under the kidney capsules of C.B-17/Icr-scid/scidJc1 (SCID) mice. As a control, whole neonatal ovary from C57BL/6 mouse was transplanted. Two weeks later, the transplants were harvested and serially sectioned for PAS staining.

### Oocyte isolation from follicles and FLSs

Ovaries from C57BL/6 mice up to 5-days-old were washed with PBS and secondary follicles were collected. The follicles or FLSs were first treated with 0.25% trypsin/0.02% EDTA for 5 minutes and then with 300 μg/ml hyaluronidase (Sigma, H3506) for a further 5 minutes. Granulosa cells were removed physically by pipetting with narrow-bore glass capillary pipettes under the microscope in an incubation chamber at 37°C. Denuded oocytes were transferred serially into medium drops four times to remove granulosa cells.

### Bisulfite genomic sequencing

Bisulfite genomic sequencing was performed using agarose gel-embedded cells as described [59] with some modifications. Twenty cells (oocytes or granulosa cells from mouse ovary, and oocyte-like cells from FLSs) were embedded in a 10 μl drop of 1.6% low-melting temperature agarose gel. The gel was treated with 100 μl of restriction solution containing 12 U *Hind*III overnight at 37°C and then denatured. After sodium bisulfite treatment at 50°C for 7 hours, followed by neutralization, TE-treated gels were utilized as PCR templates. The first round of PCR was performed using the gels (10 μl) with specific primers and LA Taq (TaKaRa, RR002A) as follows; one cycle at 94°C for 2 min; 40 cycles at 94°C for 30 sec, 50°C for 30 sec and 68°C for 1 minute; one cycle at 72°C for 7 minutes. The first round PCR products were purified with the Wizard SV Gel and PCR Clean-UP System (Promega, WI, USA, A9282) and then were used as templates for the second round of PCR. The products were purified and cloned into the pTAC-1 vector (BioDynamics Laboratory, Tokyo, Japan, DS125L) or pT7 blue vector (Novagen, CA, USA, 69820-3). Bisulfite treatment of genomic DNA from clone-G ESCs was performed with the EZ DNA Methylation-Direct Kit (Zymo Research, Irvine, USA, D5020) according to the manufacturer's protocol. Primers are shown in Table S1.

## Supporting Information

Table S1 **Primers used for RT-PCR analyses and bisulfite genomic sequencing.**
(PDF)
