# Supplementary material for: Aberrant Gene Expression and Sexually Incompatible Genomic Imprinting in Oocytes Derived from XY Mouse Embryonic Stem Cells In Vitro
Source: PLoS One. 2013 Mar 5;8(3):e58555. doi: 10.1371/journal.pone.0058555 (PMC3589367; doi:10.1371/journal.pone.0058555)
Supplement: Table S1 — Primers used for RT-PCR analyses and bisulfite genomic sequencing. (PDF) [file pone.0058555.s001.pdf]

Primers used for RT-PCR analysis

Gene	Primer name	Sequence	Product size
β-Actin	βactin-RT-S	TGGAGAAGATCTGGCACCACACCTT	225 bp
	βactin-RT-AS	CTCCGGAGTCCATCACAATGCCTGT	
BMP15	BMP15-RT-S	CATCCTTGGATGTTGCCTTC	163 bp
	BMP15-RT-AS	CAAGAGGCATCAGATTCAATG	
Boule	msBoule-RT-S	TGTTAATGACAGAGCTGGAGTGTC	600 bp
	msBoule-RT-AS	GCAATGGCACTTGAAGCATAAAC	
c-Kit	Kit-S	CCATTTGGAAAACTGGTGGT	220 bp
	Kit-AS	AGGTGAGCACCATCACAATG	
Cyp11a1	Cyp11a1-RT-S	GTGACCTTGCAGAGGTACACTGT	434 bp
	Cyp11a1-RT-AS	GTGACTCCAGCCTTCAGTTCACA	
Cyp19a1	Cyp19a1-RT-S	TAGCGCAAGATGTTCTTGGA	181 bp
	Cyp19a1-RT-AS	GCCCAATTCCCAGACAGTAG	
Dax1	Dax1 S1096	TGCTGCGGTCCAGGCCATCAAGAG	233 bp
	Dax1 AS1305	GGGCACTGTTCAGTTCAGCGGATC	
Dazl	DAZL-RT-S	TGCCAGGGACATGAGTTTTA	256 bp
	DAZL-RT-AS	CACTGAAGTAAGGGGACAAAATC	
Dmc1	Dmc1 Upper	TTCGTACTGGAAAAACTCAGCTGTATC	245 bp
	Dmc1 Lower	CTTGGCTGCGACATAATCAAGTAGCTC	
Dmrt1	Dmrt1-RT-S	TGGGTTCTGGAAGCAAGAAG	185 bp
	Dmrt1-RT-AS	CTGTCTTCTCAGGGCCACCT	
Dnmt3L	Dnmt3L-type-S	CATCTGTGAGAGCCCCGACTG	504 bp
	Dnmt3L-1061-1042	GGCAGCGCATACTGCAGGAT	
ERas	45328 S118	ACTGCCCCTCATCAGACTGCTACT	210 bp
	ERas AS304	CACTGCCTTGTACTCGGGTAGCTG	
Fgf8	Fgf8-RT-S	CAAGAGCAACGGCAAAGGCAAGGAC	246 bp
	Fgf8-RT-AS	GTGAAGGGCGGGTAGTTGAGGAA	
Figlα	Figlα-RT-S	AAATCTCAACCGTGGCTTTG	159 bp
	Figlα-RT-AS	GGGCTTTGTTTCTCTGAGACC	
Foxl2	Foxl2-RT-S2	AAATGTAATGGCCTTGGAGTTTGCT	290 bp
	Foxl2-RT-AS2	AGTGTCATCTGGGCGGTGTG	
Foxo3a	Foxo3a-RT-S	CTGTCCTATGCCGACCTGAT	159 bp
	Foxo3a-RT-AS	GTGCCGGATGGAGTTCTT	
Fshr	Fshr-RT-S	GTCATGCTCATTGAGGCCAGCCT	387 bp
	Fshr-RT-AS	TTGGCTTGTGGTCAGGACCACCA	
Gdf9	Gdf9-RT-S	GATGGTGGACCTGCTGTTTA	152 bp
	Gdf9-RT-AS	CAGAAGACATGGCCTCCTTT	
Ifitm3	msIfitm3-RT-S	GTTATCACCATTGTTAGTGTCATC	151 bp
	msIfitm3-RT-AS	AATGAGTGTTACACCTGCGTG	
Lhcgr	Lhcgr-RT-S	GCTGGAGTCCATTCAGACGCTCA	325 bp
	Lhcgr-RT-AS	AGCATCTGGTTCTGGAGTACATTG	
MIS	MIS-S	TTGGTGCTAACCGTGGACTT	319 bp
	MIS-AS	GCAGAGCACGAACCAAGCGA	
Miwi	Miwi-F	ATGATCGTGGGCATC	530 bp
	Miwi-R	AGGCCACTGCTGTCATA	
Mlh1	msMLH1-RT-S	ACCCACTTCCAGTCCAGGAAGCTC	408 bp
	msMLH1-RT-AS	CAGCCACTTTCAGGACTGTCTAAGG	
Mvh	Mvh Vg2	CCAAAAGTGACATATATACCC	417 bp
	Mvh Vas3	TTGGTTGATCAGTTCTCGAG	
Nanos2	msNanos2-RT-S	AACTTCTGCAAGCACAATGG	493 bp
	msNanos2-RT-AS	CCGAGAAGTCATCACCAG	
Nobox	Nobox-RT-S	ACAAACGCCATGAGATTTCC	215 bp
	Nobox-RT-AS	AACAGGGCCAGGTTCTAGGT	
Oct4	Oct3 U474	CTGAGGGCCAGGCAGGAGCACGAG	485 bp
	Oct3 L935	CTGTAGGGAGGGCTTCGGGCACTT	

Ptgs2	Ptgs2-RT-S	CCTGAAGCCGTACACATCATTTGA	380 bp
	Ptgs2-RT-AS	AGGCACTTGCATTGATGGTGGCT	
Sycp3	Scp3-RT-S	AACAACAAAAGATTTTTCAGCAGTCTA	236 bp
	Scp3-RT-AS	CATGGATTGAAGAGACTTTCGAACATT	
Sox9	Sox9-RT-S	AGGAAGCTGGCAGACCAGTA	193 bp
	Sox9-RT-AS	CGTTCTTCACCGACTTCCTC	
Sry	Sry-RT-S2	AAGCGCCCCATGAATGCATTTATGGT	233 bp
	Sry-RT-AS2	ACACTTTAGCCCTCCGATGAGGCTGA	
StAR	StAR-RT-S	GATTAAGGCACCAAGCTGTGCTG	239 bp
	StAR-RT-AS	CTGCTGGCTTTCCTTCTTCCAGC	
Stella	Stella-ORF-S	CACCATGGAGGAACCATCAGAGAAAGTC	457 bp
	Stella-ORF-AS	CTAATTCTTCCCGATTTTCGCATTCT	
Stra8	Stra8-RT-S	TTTGACGTGGCAAGTTTCCTGGACA	254 bp
	Stra8-RT-AS	TCTCCGGCCCTCCTGCTTTACAGAT	
Zp1	Zp1-RT-S	AAGGTGCTGGATGAATTTGG	177 bp
	Zp1-RT-AS	GGGTAGGACGGCTTGTATGA	
Zp3	Zp3-RT-S	GGGTGCAGATGACGAAAGAT	155 bp
	Zp3-RT-AS	GATAGGGTGGCTGCTCACAT	

Primers used for bisulfite genomic sequencing

Gene	Primer name	Sequence	Product size
Snrpn (1st PCR)	Snrpn-DMR sense (F1)	TATGTAATATGATATAGTTTAGAAATTAG	420 bp
	Snrpn-DMR antisense (R1)	AATAAACCCAAATCTAAAATATTTTAATC	
Snrpn (2nd	Snrpn-DMR sense (F2)	AATTTGTGTGATGTTTGTAATTATTTGG	
	Snrpn-DMR antisense (R2)	ATAAAATACACTTTCACTACTAAAATCC	
H19 (1st PCR)	H19-DMR sense (F1)	GAGTATTTAGGAGGTATAAGAATT	423 bp
	H19-DMR antisense (R1)	ATCAAAAACTAACATAAACCCCT	
H19 (2nd	H19-DMR sense (F2)	GTAAGGAGATTATGTTTATTTTTGG	
	H19-DMR antisense (R2)	CCTCATTAATCCCATAACTAT	
